# Towards improving edge quality using combinatorial optimization and a novel skeletonize algorithm

**DOI:** 10.1186/s12880-021-00650-z

**Published:** 2021-08-05

**Authors:** Marvin Arnold, Stefanie Speidel, Georges Hattab

**Affiliations:** 1 Division of Translational Surgical Oncology (TSO), National Center for Tumor Diseases (NCT/UCC) Dresden, Fetcherstr. 74, 01039 Dresden, Germany; 2 Department of Mathematics and Computer Science, University of Marburg, Hans-Meerwein-Str. 6, 35032 Marburg, Germany

**Keywords:** Edge detection, Skeletonize algorithm, Computational optimization, Post-processing

## Abstract

**Background:**

Object detection and image segmentation of regions of interest provide the foundation for numerous pipelines across disciplines. Robust and accurate computer vision methods are needed to properly solve image-based tasks. Multiple algorithms have been developed to solely detect edges in images. Constrained to the problem of creating a thin, one-pixel wide, edge from a predicted object boundary, we require an algorithm that removes pixels while preserving the topology. Thanks to skeletonize algorithms, an object boundary is transformed into an edge; contrasting uncertainty with exact positions.

**Methods:**

To extract edges from boundaries generated from different algorithms, we present a computational pipeline that relies on: a novel skeletonize algorithm, a non-exhaustive discrete parameter search to find the optimal parameter combination of a specific post-processing pipeline, and an extensive evaluation using three data sets from the medical and natural image domains (kidney boundaries, NYU-Depth V2, BSDS 500). While the skeletonize algorithm was compared to classical topological skeletons, the validity of our post-processing algorithm was evaluated by integrating the original post-processing methods from six different works.

**Results:**

Using the state of the art metrics, precision and recall based Signed Distance Error (SDE) and the Intersection over Union bounding box (IOU-box), our results indicate that the SDE metric for these edges is improved up to 2.3 times.

**Conclusions:**

Our work provides guidance for parameter tuning and algorithm selection in the post-processing of predicted object boundaries.

## Background

Boundaries are crucial in object detection methods. They provide the means to distinguish objects of interest in an image from the background. In humans, this task appears to be intuitive although it is accomplished using complex processes that integrate *a priori* knowledge. Knowing where the boundary of an object lies requires human annotators and/or algorithms to find a dividing line between two areas of an image that exhibit different visual features (e.g., object boundaries, material properties boundaries, lighting boundaries). However, in both cases there remains an uncertainty as to where the edge lies. This uncertainty varies and is even exacerbated by the domain specificity (e.g., natural image domain). In turn, it prevents the definition of precise edges and the generalization of edge-related methods. In this work, we distinguish between this area of uncertainty (ramp edge) and a thin edge. Our problem description defines the former as the boundary, where the intensity change is not instantaneous but occurs over a finite distance with the occurrence of discontinuities (i.e., incomplete object boundary). The latter, or thin edge, is a 1 pixel wide exact representation from a post-processed ramp edge. While a boundary in an image is represented as a per-pixel uncertainty value, an edge is represented as a per-pixel binary dichotomy. Our aim is to improve the creation and definition of ramp edges after they have been detected to meet the constraints of an 1-pixel edge. That is to say, post-processing the ramp edge, i.e., the boundary, to a thin edge.

Historically, edge detection is a core discipline in computer vision that allows many different tasks, e.g., image segmentation [1], object detection [2, 3], and augmented reality [4]. These tasks have evolved in different directions: (a) pixel based, where individual pixel value differences are investigated in a local neighborhood of an edge [5–7], (b) models based on human perception [8], and (c) computational approaches on step edges [9]. However, these works provide all edges of an image and the task of filtering out non-relevant edge remains. The extraction of task-relevant edges, called semantic edges, is an active research topic across many research fields [10–14]. A graph-based solution was introduced by stitching together appropriate edge fragments to produce a closed or linked object boundary [15]. Boundary fragments are identified using an encoding of the Gestalt laws of proximity and continuity. Two other works were proposed that rely on the concept of object consistency. They tackle the same task of segmentation, however they do so by considering hue or saturation between neighboring objects. One proposed solution used the color differences of neighboring pixels combined with progressive block-oriented edge detection [16]. Another solution introduced the use of differences in gray values between pixels with the addition of not taking into account edges between other features [17].

In the medical research field, deep learning based algorithms have been reported [10, 14, 18, 19]. For minimally invasive surgery, and specifically laparoscopic image data, the task of semantic segmentation has proven challenging due to different limiting factors (e.g., the occlusion by fatty tissue or surgical instruments, or the homogeneity of the texture of the target organ) [20]. In our proposed deep learning based edge detector [14], the neural net predicts kidney boundaries that are then post-processed into 1 pixel wide edges. In this work, the specific edge location is determined by a confidence value, that is represented by the pixel intensity within the predicted boundary. Related work relied on this same approach to address the task of head-circumference delineation from fetal ultrasound images in the domain knowledge of prenatal ultrasounds [21]. Yet, adapting this algorithm warrants further investigations to improve post-processing steps.

This work details the how to improve the resulting edges by proposing a formalization of an edge detection pipeline that contains the conversion of such boundaries or ramp edges to thin edges as a post-processing step. This allows us to seamlessly integrate our approach into many existing edge detection pipelines and help improve methods for a variety of tasks. Further, we introduce a novel skeletonize algorithm and evaluate its performance compared to the state-of-the-art results across different research fields (c.f., Table 3). We benchmark our algorithm against morphological medial axis transformation algorithms to enable a comparable evaluation. We consider these three algorithms: a 2D skeletonize [22], a 3D skeletonize [23], and the novel Gray-Weighted Path Skeletonize (GWPS). To evaluate and improve the accuracy of the resulting edges, we use state-of-the-art metrics, that is the Signed Distance Error (SDE) and the Intersection over the Union of the bounded connected component (IoU-box), respectively. To avoid introducing and separating metrics of downstream tasks from edge-specific metrics, we restrict our analyses to the resulting 1-pixel edge maps. We assess the validity of the proposed post-processing algorithm by relying on three data sets: (1) Kidney boundaries [14], (2) NYU Depth Dataset V2 [24], and (3) BSDS 500 [25].

This work would help the community at large by presenting: (a) a parameterized post-processing algorithm to achieve a thin edge that also enables a combinatorial optimization, (b) a novel skeletonize algorithm by intermediate transformation into a gray-weighted distance image, and (c) an extensive evaluation regarding performance metrics.

## Methods

To improve the accuracy of topological skeletons, we first formalize an edge detection pipeline as a composition of multiple functions. Second, and to enable a combinatorial optimization, we describe the post-processing function, including its parameters. Third, we propose a novel skeletonize algorithm that is adapted to better object boundary processing. Fourth and last, we detail the used data sets, lay out the evaluation, and report the employed metrics.

We define an edge detection pipeline ${\mathcal{P}}$ as a composition of two functions:

1 $$\begin{aligned} {\mathcal{E}} \circ {\mathcal{A}}: I^3 \rightarrow I^B, \quad x \mapsto ({\mathcal{E}} \circ {\mathcal{A}})(x; \alpha , \beta , \gamma ), \end{aligned}$$

where $I^3$ is the space of the three RGB channels and $I^B$ the space of binary images. The function defining the pipeline contains two parts:

2 $$\begin{aligned} {\mathcal{A}}: I^3 \rightarrow I^1,\quad x \mapsto {\mathcal{A}}(x), \end{aligned}$$

where $I^1$ is the space of 1-channel gray scale images.

3 $$\begin{aligned} {\mathcal{E}}: I^1 \rightarrow I^B,\quad x \mapsto {\mathcal{E}}(x; \alpha , \beta , \gamma ). \end{aligned}$$

This function composition maps our approach of predicting ramp edges or boundaries, then processing them into thin edges as follows:

1. ${\mathcal{A}}$ takes a RGB image $s \in S$ as input and predicts the boundaries of the objects of interest as a gray scale image $g \in G$.
2. ${\mathcal{E}}$ extracts from $g \in G$ the thin edges and produces the output of the pipeline $o \in O$, i.e., binary image.

**Fig. 1:**
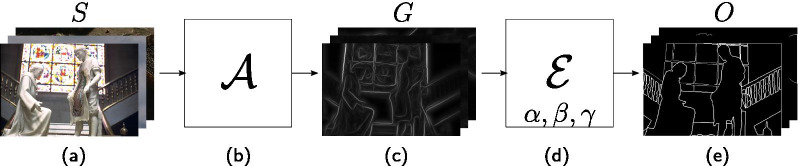
Edge detection pipeline $\varvec{\mathcal{P}}$. The pipeline is split into five parts: (**a**) $S$, the set of input images in RGB color space, (**b**) the function ${\mathcal{A}}$ that takes $G$ as input and produces a boundary image set $S$, (**c**) the output of ${\mathcal{A}}$ as gray scale images, (**d**) the edge extraction function ${\mathcal{E}}$ with parameters $\{\alpha , \beta , \gamma \}$ that converts boundaries into edges, (**e**) the output $O$ of the function ${\mathcal{E}}$, the set of binary images containing thin edges. Figure 3 shows a specific example this pipeline

*S*, *G* and *O* are sets of images in image spaces $I^3, I^1$ and $I^B$, respectively. Figure 1 presents a visual overview of ${\mathcal{P}}$.

### Edge extraction function ${\mathcal{E}}$

The predicted boundaries of an image $g \in G$ from ${\mathcal{A}}$ are converted to a thin edge using the extraction function ${\mathcal{E}}$ defined in Eq. 3 with the following parameters:

1. *Input image* $g$: Gray scale output image of function ${\mathcal{A}}$ containing the predicted boundaries.
2. *Threshold* $\alpha$: Due to noise in the predicted boundary, we set a pixel value threshold $\alpha$. Iff the pixel value is below $\alpha$, the pixel gets set to a value of zero, otherwise it remains the same.
3. *Skeletonize algorithm* $\beta$: This algorithm converts the boundary to a thin edge. We benchmark our skeletonize algorithm ‘GWPS’ to the following algorithms: 2D skeletonize [22] (called ‘2D’), 3D skeletonize [23] (called ‘3D’). For the ‘2D’ and ‘3D’ algorithm the boundary images are converted to binary image representations before applying the skeletonize algorithm. That is to say, while a pixel value of 1 implies a contribution to the boundary, 0 belongs to the background.
4. *Pruning* $\gamma$: After applying the skeletonize algorithm spurs are introduced because of small irregularities in the boundary. Spur pruning removes all spurs with a maximum length $l$ [26]:
  4 $$\begin{aligned} l = \frac{\gamma }{100}*\sqrt{w^2+h^2}. \end{aligned}$$
  With the image width $w$ and height $h$ in pixels. This definition allows for the relative comparison of the pruning effectiveness between images of different sizes.

Our post-processing algorithm (Algorithm 1) defines the necessary steps to implement the aforementioned edge extraction function ${\mathcal{E}}$ on a per image basis. 
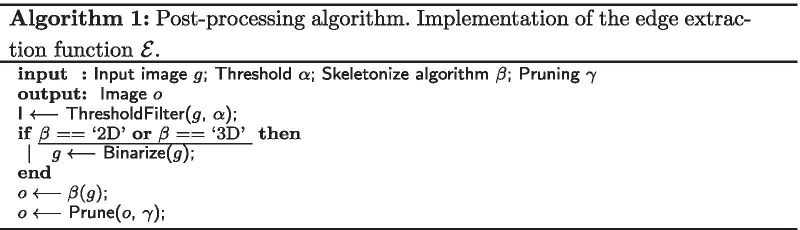


The parameter set $\{\alpha , \beta , \gamma \}$ is bounded, such that:

5 $$\begin{aligned} 0\le &\alpha \le 255, \\ &\beta\in \{\text{'2D'}, \text{'3D'}, \text{'GWPS'}\}, \\ 0\le &\gamma \le 100 \end{aligned}$$

#### Gray-weighted path skeletonize (GWPS)

**Fig. 2:**
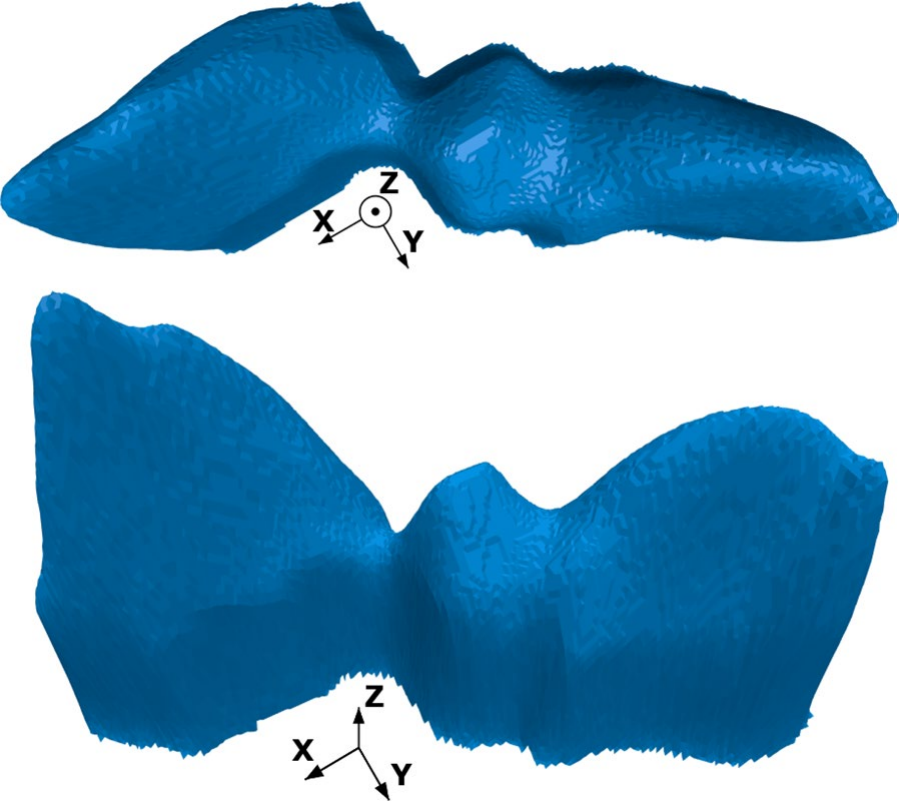
Three-dimensional representations of a predicted boundary. Images created by mapping the increasing pixel intensity to height. Algorithm 2 retrieves the ridge of the three-dimensional surface as a thin edge in two-dimensional space

Figure 2 shows an example predicted boundary as a three-dimensional surface. The goal of the GWPS algorithm is to find the ridge along this surface and extract it as a thin edge in two-dimensional space. To use the ridge of the surface, we introduce an algorithm that computes the resulting edge with the help of an intermediate representation of the distance field using a Gray-Weighted Distance Transformation or GWDT [27]. The GWDT assigns each contributing pixel a value representing the cost of the shortest path to the background, where costs correspond to the accumulated pixel intensity values along the path. In the context of our problem, background pixels have the value zero and therefore all other non-zero pixels are contributing information to the thin edge.

The GWDT value at pixel position (*i*, *j*), given an image; where *f*(*i*, *j*) represents the pixel value at position (*i*, *j*), is calculated by minimizing the accumulated cost along the path from the current position to a background pixel. A pixel path *W* is defined as

6 $$\begin{aligned} W = ((i_1, j_1), (i_2, j_2), \ldots , (i_n, j_n)) \quad n \in {\mathbb{N}}_{\ge 2} \end{aligned}$$

where the following properties hold:

7 $$\begin{aligned} \left\vert i_r - i_{r-1}\right\vert + \left\vert j_r - j_{r-1}\right\vert &= 1, \\ f(i_1, j_1), \ldots , f(i_{n-1}, j_{n-1}) &> 0, \\ f(i_n, j_n) &= 0 \end{aligned}$$

That is to say, all pixels in the sequence are four-way connected, the first $n-1$ pixels are foreground pixels, and the path ends in a background pixel. We optimize the cost along this path for all non-zero pixels of the input image and assign the cost as the corresponding pixel value of the GWDT image $t \in I^1$

8 $$\begin{aligned} t[i, j] = \min _{W} \sum _{(i_r, j_r) \in W}f(i_r, j_r), \end{aligned}$$

that is to say, the pixel value at position (*i*, *j*) is optimal as it is set to the minimum of the accumulated costs along the pixel path *W*.

Using the resulting image as an intermediate representation, we propose Algorithm 2 to reduce the GWDT to a thin edge. This algorithm uses the eight-way neighborhood number $N_c$ [28] that is defined for a pixel $x_0$ using its neighborhood, as depicted in Table 1 and Eq. 9.

**Table 1:**
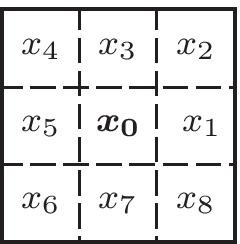
Eight-way-neighborhood numbering around center point $x_0$ [28]

9 $$\begin{aligned} N_c(x_0)&= \sum_{k\in \{1,3,5,7\}}{\overline{x}}_k-{\overline{x}}_k*{\overline{x}}_{k+1}*{\overline{x}}_{k+2} \\ {\overline{x}}&= 1-x \end{aligned}$$

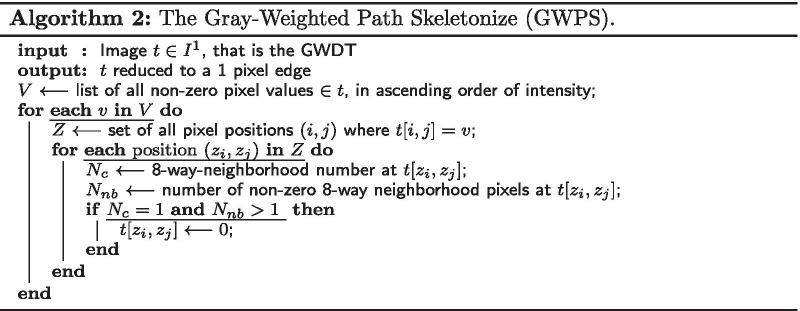


The resulting image contains the desired thin edge represented by non-zero pixels along the ridge of the distance field’s surface. The edge is also eight-way connected due to the evaluation of the eight-way-neighborhood number. In summary, our skeletonize algorithm comprises two steps:

1. Calculate the GWDT of the image and,
2. Extract the thin edge of the GWDT by running GWPS (c.f., Algorithm 2).

### Data sets

To meet the requirement of creating thin edges, the pipeline is used to find the best parameter sets for the post-processing methods. This means the evaluation is carried out for every data set on the predicted boundaries versus the published edges. We report below the data sets that are used to evaluate our computational strategies and the pipeline usage:

- *Kidney boundaries:* We use this data set as is [14]. It consists of 2250 images captured during porcine partial nephrectomies and for the task of kidney edge detection. For the investigated method, we do not modify the original pipeline.
- *NYU Depth V2:* We adapt this data set to an edge detection problem [24]. This is achieved by using pre-processing scripts from [29] that extract edges from ground truth segmentations. The data set contains 1449 images from a variety of indoor image scenes. For the two investigated methods, we only use the RGB image components. That is to say, we disregard the depth component.
- *BSDS 500:* We use this data set as is. It contains natural images with human ground truth object boundary annotations. This data set contains 500 images [25]. For the three investigated methods, we do not modify the original pipeline.

### Evaluation

To improve the resulting thin edges, both qualitatively and quantitatively, we focus on the metrics as reported in the state of the art [14]. Specifically, metrics that reflect the reliability of all evaluated pipelines. That is to say, the SDE and IoU-box metric, as the former integrates both precision and recall, and the latter evaluates the thin edge shape and its absolute position to the ground truth.

To find the optimal parameters for function ${\mathcal{E}}$, we perform a combinatorial optimization iteratively for each data set; that is on the validation set. The parameter combination yielding the best SDE result is then selected and used for evaluation of the test set. The parameter set of function ${\mathcal{E}}$ is evaluated in the bounds defined by Eq. 5. The originally presented pixel accuracy based metrics (i.e., precision, recall and SDE) are modified to be used in cases where no edges are predicted. Yet, some are present in the corresponding ground truth image and vice versa. This is modification is achieved by identifying all described cases during evaluation and then adding an edge pixel in the center of the empty binary image.

## Results

**Fig. 3:**
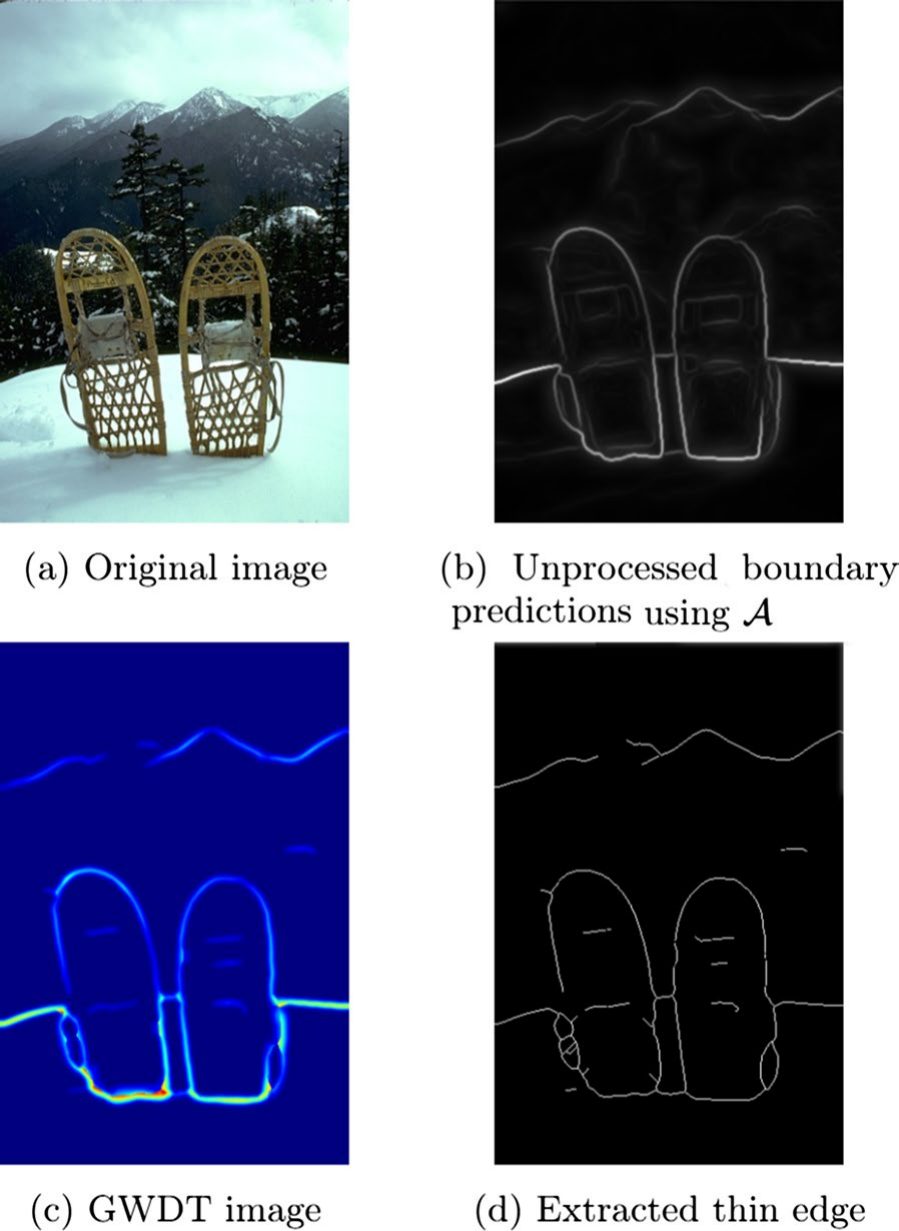
Example illustration of an edge detection using $\alpha =40, \beta =\text{'GWPS'}, \gamma =2$ of one sample image in the BSDS 500 data set and the work of [25]. The boundaries of the original image (**a**) are shown in (**b**). By performing a GWDT as described in Eq. 8, we obtain (**c**). After applying the skeletonize algorithm (c.f., Algorithm 2), we obtain the thin edge (**d**)

We present the results for the parameter search regarding the function ${\mathcal{E}}$, then compare our results on the test sets of each related work. Figure 3 shows the result of a sample image at different parts of our pipeline.

In Table 2, we found that in four of the six investigated related work (66.67%), the edge extraction yields the lowest SDE score by using the GWPS algorithm. Further, pruning was only necessary in one pipeline, as all other reported parameter combinations are optimal with $\gamma = 0$. That is to say, pruning was not used.

**Table 2:** Parameter sets found during validation of the combinatorial optimization using Algorithm 1

Data set	Work	$\alpha$	$\beta$	$\gamma$	*SDE*
Kidney boundaries	[14]	240	2D	0	1144.7
NYU Depth Dataset V2	[29]	100	2D or 3D	0	4.2
	[31]	40	GWPS	0	7.4
BSDS 500	[25]	40	GWPS	2	24.5
	[32]	160	GWPS	0	16.0
	[33]	190	GWPS	0	10.6

The optimal parameter set $\{\alpha , \beta , \gamma \}$ minimizes the mean *SDE* metric

We evaluated the best parameters on the test part of each data set. All results are reported in Table 3. The term original refers to running the methodology as is, meaning that we follow the original data set usage and post-processing algorithm. We report in parenthesis the standard deviation for each result.

**Table 3:** Evaluation results using optimal parameters on the test part of each data set

Data set	Work	Post-processing	*SDE*	${\varvec{\tilde{x}}_{\varvec{SDE}}}$	IoU-box	${\tilde{\varvec{x}}_{{{\mathbf{IOU-{box}}}}}}$
Kidney boundaries	[14]$^{\text{b}}$	Original$^{\text{d}}$	232.9	103.5	0.32	0.33
		Our method	2515.0	954.5	0.04	0.02
NYU depth dataset V2	[29]$^{\text{a}}$	Original	4.9	4.3	0.6	0.6
		Our method	4.2	3.8	0.6	0.6
	[31]$^{\text{a}}$	Original	8.1	7.3	0.9	1
		Our method	7.4	6.4	1	1
BSDS 500	[25]$^{\text{a}}$	Original	50.0	25.6	0.8	0.8
		Our method	21.8	12.0	0.6	0.6
	[32]$^{\text{c}}$	Original	11.1	7.9	0.5	0.6
		Our method	12.0	6.8	0.8	0.8
	[33]$^{\text{c}}$	Original	9.5	7.1	0.5	0.4
		Our method	10.3	6.3	0.8	0.9

Reported metrics are mean and median values (${\tilde{x}}$). We compare the results of Algorithm 1 to each method specific results. We note that the original post-processing implementations did not include a thresholding step except for [14]

Originally used post-processing methods: $^{\text{a}}$ 2D skeletonize algorithm, $^{\text{b}}$ 3D skeletonize algorithm, $^{\text{c}}$ standard non-maximum suppression. $^{\text{d}}$ Values in this row were taken from the original paper and not recomputed (c.f., discussion section point three for details)

Using our previous work [14], we achieved an SDE score of 2515.0 ($\sigma = 7053.22$), which is 10.8$\times$ worse than the original, and an IoU-Box of 0.04 ($\sigma = 0.07$).

Using the NYU Depth Dataset V2, our Algorithm 1 achieved a SDE of 4.2 ($\sigma = 1.7$) and an IoU-Box of 0.6 ($\sigma = 0.2$). However, using the algorithm of [30], we achieved a SDE score of 7.4 ($\sigma = 3.91$) and an IoU-Box of 1 ($\sigma = 0.04$) using [31]. For this data set, we improved the average SDE by 1.13 times (x).

Using the BSDS 500 data set and the algorithm of [25], we reported a SDE score of 21.8 ($\sigma = 31.28$). That is an improvement of 2.3$\times$, while maintaining a similar IoU-Box score of 0.6 ($\sigma = 0.18$). While we increased the SDE using the algorithm of [32] to 12.0 ($\sigma = 13.08$), that is 1.08$\times$ worse, we managed to increase the IoU-Box to 0.8 ($\sigma = 0.17$), that is an 1.6$\times$ increase. Using the algorithm of [33] an 1.08$\times$ increase in SDE to 10.3 ($\sigma =9.51$) was reported. Although, increasing the IOU-Box to 0.8 ($\sigma =0.15$), which corresponds to an improvement of 1.6$\times$.

## Discussion

Our evaluation of a new and refined post-processing algorithm have found improvements, in regards to the average SDE and the IOU-box metrics, across several data sets and the majority of the established works. Indeed, using an optimized parameter set for our proposed edge detection pipeline ${\mathcal{P}}$, specifically for the function ${\mathcal{E}}$, was found to be beneficial to improving the measured edge quality. Instead of relying solely on fixed classical post-processing algorithms, our work proposes adjusting the algorithm to adopt the parameter set to the task at hand. We discuss several pointers below.

First, to find the optimal parameter combination we used a non-exhaustive discrete parameter search. Due to the computational expense of such a parameter search, we reduces the search space by evaluating every tenth value for $\alpha$ and each integer from 0 to 10 with the addition of every tenth value from 20 to 100 for $\gamma$, respectively. Although computationally intensive, the granularity of such a search may be increased to find better sets of suitable parameters for each tested work.

Second, the introduction of an error correction step, that is to say pruning, was determined to only improve the evaluated metrics for one of the examined works. Evaluating the pruning with a higher granularity may lead to a potential improvement of metrics by allowing non-integer pruning values.

Third, the modification of the SDE computation (c.f., evaluation section) led to an inconsistency in the evaluation of our previous work [14]. Due to predicted images with no edge, the original evaluation algorithm miscalculates the SDE value. By implementing our adjusted SDE calculation algorithm, we obtained higher SDE scores for a few predicted images. This can be seen in the discrepancy between the mean and median value as well as the high standard deviation of 7053.22. This is mainly due to the post-processing algorithm failing on a small subset of the images.

Fourth, the usage of a boundary that represents a distance field, that is used in [14], prompted us into creating Algorithm 2. Using the available certainty information inside the boundary, our algorithm is designed to find the optimal edge along the ridge of the three-dimensional representation of the step edge or the boundary. Although the algorithms from the investigated works were not particularly designed with this idea in mind, we apply the GWPS algorithm on all boundary based predictions as presented in Table 3. Additionally, we hypothesize that our results may be improved if we incorporate the distance field based prediction into the respective algorithms. For example, this could be achieved by training a neural network to make the predicted boundaries more suitable for Algorithm 2.

Fifth, the choice of the $\alpha$ parameter is critical to the quality of the resulting 1-pixel edges. The presence of noise in the input data to our pipeline is a major factor in poor edge predictions. Existing methods that adapt our pipeline as a post-processing step should take special care in setting the $\alpha$ parameter, as it reflects only a very rudimentary form of noise filtering. Rather, it might even be advantageous to use additional noise reduction to achieve better results. The influence of the $\beta$ parameter is less than that of $\alpha$, as tests have shown similar but only slightly worse results when the selected $\beta$ is changed in Table 3. $\gamma$ influences the results the least, as seen from Table 3, and can often be neglected.

Sixth, our evaluation pointed towards a complex interdependence between the employed boundary prediction algorithm, including edge detectors, the data set, and the resulting optimal parameter choice. To achieve the best SDE score, we conducted a sensitivity analysis. This permitted us to find the most suitable parameter set for the used data sets. In the worst case scenario, the combination of other data sets, computational approaches (e.g, detectors, predictors, etc) and the wrong parameters, would result in unsatisfactory results. Given these combinations, we recommend additional investigation of the failing pipeline step and the implementation of countermeasures, e.g., the noise reduction step (see previous discussion point). Given an example data set and two algorithms, we found that the difference in value of each parameter pair influenced our decision for a good post-processing algorithm. Although this decision is non-trivial, formalizing an evaluation pipeline from end-to-end better helped us consider the solution space and find the optimal one. In sum, the evaluation results support our effort to improve the resulting edges.

Seventh, the choice of an appropriate post-processing algorithm is an important step in the definition of an edge detection method. Yet, the related work that tackles the task of edge detection are not attributing the necessary care of parameter tuning and algorithm selection regarding the processing of the predicted boundaries. We believe that an integration of our parameter optimization approach will help achieve a better edge quality.

Eighth, the evaluation of additional data sets, such as the PASCAL VOC 2012 [34] or Multi-cue [35] data set, could provide insight into the robustness of our proposed method in a greater variety of domains. Unfortunately, due to the unavailability of ground truth data for the PASCAL VOC 2012 data set and computational limitations this remains part of a potential future work.

Ninth and last, regardless of the application area, the task of edge detection can be supplemented by our herein presented methodology to optimize the post-processing step. This work can be generalized using the following two steps: (a). evaluate the SDE metric on the validation set using an appropriate parameter search technique and find the optimal values for $\{\alpha , \beta , \gamma \}$, and (b). use this parameter set and the selected algorithms for post-processing the test set data. We examined different approaches and their corresponding parameters of step (a) and (b) and can therefore provide a reference to adapt our post-processing algorithm. We believe that our work is a stepping stone for future endeavors that involve edge detection tasks using predicted signed distance fields or boundaries in images.

## Conclusion

In this work, we have introduced a post-processing algorithm that can be employed in an edge detection context. Despite the high impact on edge quality, edge detection methods primarily focus on one specific post-processing reasoning. We investigated how edge quality can be improved by introducing a post-processing algorithm with multiple parameters. By incorporating the parameters of thresholding, the type of the skeletonize algorithm, and the pruning into an edge extraction function, we were able to improve state-of-the-art metrics in multiple pipelines, across different data sets and from multiple domains. The division of the evaluation process into multiple parameters and making our work open source renders our approach readily available, adaptable, and extendable. Additionally, we introduced a novel skeletonize algorithm that works in analogy to extracting the ridge of a 3D surface and projecting it into a 2D plane. Apart from the challenge of improving kidney boundary edge detection, our pipeline successfully improved SDE or IoU-Box metrics for all other tested works on the two different data sets. Based on our evaluation pipeline, we believe that future edge detection methods can achieve better edge quality.

## Data Availability

The datasets analysed during the current study are available at https://endovissub2017-kidneyboundarydetection.grand-challenge.org/ (kidney boundary, on request), https://cs.nyu.edu/~silberman/datasets/nyu_depth_v2.html (NYU-Depth V2, public) and https://www2.eecs.berkeley.edu/Research/Projects/CS/vision/grouping/resources.html (BSDS 500, public). All evaluated results and source code is publicly available in the edges repository at https://github.com/fuxxel/edges.
